# A Clinical Lecture on the Use of Calomel in Remittent Fever

**Published:** 1849-11

**Authors:** 


*A Clinical Lecture on the Use of Calomel in Remittent Fever.* By W. G. Ramsay, M. D.

The next remedy which will occupy our attention is *Mercury*—and I wish you to give your attention seriously to the subject—as the whole pathology of fever rests here. The action of this remedy has been one of the principal causes of the great changes and uncertainty in the treatment of fevers. Its inordinate administration in almost all diseases is entirely the effect of a mistaken pathology. It is necessary that we should recur to what we have said when treating of the Pathology of fever. We said we believed, with many, that the primary impression of fever was on the gastro-intestinal canal, and that the liver was secondarily affected, and not primarily, as was thought by many physicians. We proved to you that the bilious vomiting in fever was caused by an affection of the stomach, such as could be produced by sea-sickness, or the action of an emetic in a healthy person. To corroborate more fully these opinions, I have examined many of the old works on fever, which have been much overlooked, and I find I am substantiated in what I say by many authors, although it is said by many at the present time—"that attributing fever to the stomach, is something new and unheard of." Fordyce, in his work on fever, remarks:—

"It has often been supposed that the redundance of bile constituted an essential part of an attack of fever, whereas it is mere accident. If the pancreatic juice had been blue, and had any particular taste or smell, and the bile had been colorless, insipid and inodorous, or as much so as the pancreatic juice is, in that case whatever has been said of the redundancy of bile as an essential part of the attack of fever, would have been said of the pancreatic juice. The bile thrown out is the consequence of the sickness, exactly in the same manner as it is the consequence of the sickness arising from the agitation of a ship at sea, and is not to be taken further than as a mere accident in the attack of fever. The loss of appetite increases, and the nausea and vomiting take place so instantly at the beginning of fevers that they can hardly be considered otherwise than as an affection of the stomach itself."

John Hunter, in his observations on the diseases of the Army in Jamaica, remarks:—

"The discharge of bile in the vomiting of remittent fevers is evidently the effect and not the cause of the fever. The stomach and bowels suffering severely in remittent fevers, communicate a degree of irritation to the liver—and the same thing happens in the operation of strong emetics and in sea-sickness, and thence a copious discharge of bile.

"The dark brown color of the bile, and its ropy consistence, are nothing more than natural changes produced by its stagnating for some time in the gall bladder and biliary ducts. The thinner parts of the bile are absorbed, and what remains becomes of a deeper color and of a thicker consistence, as happens in other secretions."

Dr. Currie, in his observations on bilious fever, published in 1728, also remarks:—

"In some cases at the beginning of fever, sickness at the stomach, accompanied with vomiting and discharges of bile from the bowels, is so predominant, especially in children whose systems are extremely irritable during the season when remittents prevail, in consequence of the summer's heat, and often from the effects of teething, that many physicians have supposed that a redundancy, or a depraved condition of that fluid was the principal cause of the fever, and in infants it has been confounded with cholera-infantum. This is, however, a great error, for vomiting, by whatever means excited, if often repeated with violent efforts, scarcely ever fails of forcing bile from the biliary ducts, and sometimes in enormous quantities. An extraordinary secretion of bile in this fever and in every other, is the effect of great irritability of the stomach, in conjunction with a defect of power in the circulating vessels on the surface of the body, the consequence of which is a preternatural determination to the vena cava, liver, &c., and of course the secretion of an extraordinary quantity of bile in a given time."

Bancroft in his essay on yellow fever, published in 1811—when speaking on this subject, remarks:—

"Nausea, followed by frequent vomiting, even in the early part of the disease, and by the discharge of a considerable quantity of bile, a discharge which appears in these cases to be caused solely by the efforts to vomit, in the same manner as in sea-sickness and other affections in which the evacuation of bile, and even the increased secretion of that fluid (which violent vomitings are believed to occasion) are merely a consequence and not a cause of the disorder."

"These indications of a morbid affection of the stomach in yellow fever are accompanied with others, affording similar evidence of the state of that organ, particularly excessive thirst, acute pain, and burning heat in the stomach, with so great a degree of tension and soreness over the epigastric region, that external pressure cannot be endured."

It is not necessary for me to cite more authority than I have done, to prove to you that the bilious vomiting in fevers does not indicate a disease of the liver, nor is it the cause of all the evils attending fevers, as is thought by many persons, who, entertaining such erroneous opinions, have directed their whole attention at relieving this supposed disease of the liver. I might bring to my aid the whole of the pathologists of the present day, to prove this point—but the facts are so evident to every reflecting mind, that I deem it unnecessary.

I have premised these few observations before speaking of the action of mercury and its use in fevers. As it is necessary to understand the cause of the bilious discharges, for which symptom mercury has been always exhibited, to correct, as is said, this "vitiated secretion," and restore the healthy action of the liver. The stomach, duodenum and liver are intimately associated, by means of the cœliac, hepatic and solar plexuses of the ganglionic nerves. It is through the stomach and duodenum, the viscera directly and almost constantly exposed to aggression, that morbid irritations reach the liver. The bile, like the liver, is accused of causing innumerable mischiefs, and of occasioning extensive morbid derangement in the system, of which it is entirely innocent. The liver, then, I believe, is always consecutively affected in fevers. The cause producing this derangement, if exasperated or allowed to continue, will derange the natural quality of the bile. Since the stimulation felt by the liver is always through the medium of the gastro-intestinal mucous membrane, we should always endeavor to calm this latter irritation, and this precept should be considered as the basis of our treatment in restoring the secretory action to a healthy condition.

Let us now, in connection with what we have said, investigate what is known on the action of mercury, and how it is used as the "*sine qua non*" in the treatment of fevers. This is the only way to arrive at any thing like truth in our inquiries. Its mode of action is seldom spoken of by its advocates. You only hear that if you can salivate a patient he cannot die. But this is a fatal error—much mischief is too often done before this curative indication can be effected. Mercury acts on the animal economy in two ways: it has first, a local operation on the parts to which it is applied, which is stimulant; its second operation is constitutional, affecting the glandular system by means of absorption. To prove that its primary effect is stimulating, I need but refer you to its sensible and visible action on the stomach and intestines, when administered internally. It seldom or never fails to produce nausea and vomiting, followed by purgation. This is denied by many who have studied the laws of nature so little that they can scarcely believe that vomiting is produced by irritation. Can it be denied that a dose sufficiently large will produce a cathartic effect? We have already said that catharsis is caused by the stimulant action of the article used, increasing the secretion of the intestines.

"Calomel," says Wilson Philip, "when taken internally, is doubly applied to the stomach and bowels immediately, and through the medium of the circulation, for we often have to contend with its irritating effects on the alimentary canal when it is only introduced by the skin. On the canal and on the salivary glands alone, its passage excites sensible irritation, which, if considerable, causes inflammation, in the former, only superficial, and generally in a slight degree, but in the latter often such as to affect all the neighboring parts."

In Begin's Therapeutics I find the following passage on the action of mercury:—

"The immediate action of mercury upon the stomach and intestines is soon followed by all the signs of vascular excitement, an artificial febrile excitement often appears, the pulse becomes quick, full and frequent, the heat of the skin is increased, there is a tendency to congestion of blood and to hemorrhage, the pectoral and abdominal viscera are irritated, hemorrhoids, catamenia and epistaxis come on. But the effects of this over excitement are particularly felt by the secretory organs. In the majority of cases the bile is more abundantly poured into the digestive canal, whose excitement is communicated to the liver; the quantity of pancreatic juice is increased, as also the secretions of the skin and kidneys; the salivary glands are irritated, tumefied, and their secretions considerably augmented. Applied externally, mercury is absorbed and produces the same effects, but with less violence and rapidity."

In a very valuable Essay on the therapeutical properties of Mercury, by Dr. Sacks, a Professor in a German University, I find the following passage:—

"When prescribed in small doses and continued for some time, its immediate effects are more or less stimulation of all the mucous surfaces, as the respiratory, the digestive and the genito-urinary, and soon after a similar modification of all the dermoid and glandular apparatus; the secretions are generally increased, especially those of mucous membrane of the alimentary canal, the liver and the skin. The properties of both the secreted and the exhaled fluids are considerably modified, the mucus is rendered more viscid, the urine becomes turbid, the dejections from the bowels are thinner, and of a darker or greener hue than is common; the cutaneous perspiration becomes clammy. To these symptoms are superadded others, which denote more or less gastric disturbance."

We have endeavored to prove that the first impression of the mercury is stimulant, and acts principally on the stomach, duodenum, liver and pancreas, and the excitement which it produces on these organs causes them to pour out their secretions through the means of their ducts. A large dose of calomel makes an impression which is felt by the whole alimentary canal, and a cathartic operation is produced. In small doses the action is more irritating, because it does not pass off by the bowels, but each succeeding dose adds to the irritation already existing. This, then, accounts for the difference between the effects of large and small doses of calomel.

There is yet another action of mercury to which I have not alluded, I mean its alterative action when employed in very small doses, the diminished energy of its power brings it within the limit of physiological or healthy action—the functions are not deranged—or threatened with disorganization by the violence of its operation. No immediate appreciable action is to be discovered. It acts slowly but safely. The *local* action never destroys its *specific* action.

Let us apply what we have said to the action of mercury, to its use, and the purposes for which it is given in the treatment of what is commonly called "Bilious Fever." This medicine is more vaguely employed by professional men than any article of the Materia Medica—which accounts for the popular opinion that nothing is known of its "*modus operandi.*" In fevers, when there is bilious vomiting, this remedy is always resorted to by some of its advocates, for the purpose, it is said, of restoring the secretions, by others, to promote the secretions, and by many, because they hear that it has been beneficial in fevers. Now, we have proved to you that the bilious ejections are produced by the impression of the morbific cause on the stomach, which produces vomiting, and which circumstance causes the liver to pour out its secretions, as any emetic substance would do.

Pathology has revealed to us, without the shadow of a doubt, that when the stomach is irritated, the irritation is quickly transmitted to the duodenum, liver and pancreas. What then would be the most natural way of arresting these inordinate secretions. Could you, in reason, do it safely and effectually by administering any medicine whose known action is on the stomach and duodenum? Can you destroy an effect by increasing the cause? No, gentlemen, if your knowledge of diseases is built on sound pathological principles, you would directly endeavor to remove the cause, and the effect will vanish. You would relieve the affection of the stomach, and the bilious vomiting will cease. Not by giving large and repeated doses of "calomel" to act on the liver, as is said, (for you must recollect that your medicine has a great and important organ to act upon before you can have what is known by "the specific action of mercury on the liver," as if it could immediately reach the liver without its impression being felt by every other part with which it must come in contact) but by the remedies which we have already spoken of, as calculated to relieve the inflammation of the stomach. If you had never heard of the use of calomel in bilious fever, and had studied all the phenomena of the disease, without being prejudiced by the precepts of your books and teachers—which too often make a lasting impression on your inexperienced minds, when you are incapable of judging for yourselves on the pathological truths of their operations—you could easily conceive that fever can be successfully treated without the administration of this medicine. But you retain them as established facts, without ever stopping to reason with yourselves of their correctness, and when you have commenced practice, these opinions, which but yesterday may have made but a slight impression on your minds, are now so indelibly fixed, that you will find it almost impossible to eradicate them; they are, as it were, a second nature. I say, then, if practitioners were not so much guided by the authority of others, to form the plan of their treatment, they would adopt another and a more rational mode of rectifying the evil. There has always existed a class of practitioners who reject all reasoning on the therapeutical action of medicinal agents, and their relation with the parts of the human body with which they come in contact, they have a particular set of medicines which they have been in the habit of prescribing, and nothing can change them from their accustomed routine, and they have a certain set of phrases to explain the action of their remedies. This is fully verified in the exhibition of the article under consideration. We have already considered one of their most fashionable terms, and showed how perfectly at variance it is with all physiological laws. Another of their terms is, that calomel is given to restore the healthy action of the liver. I have shown you that the great error which practitioners have been led into on this point, is caused by their believing that the liver is the primary seat of fever. When, if they would pay the slightest attention to a case of fever, they would see that all the secretions are arrested. Even allowing the liver to be the sole seat of fever, and its secretion vitiated by disease, how can you expect to change this vitiated secretion of the liver by irritating the gland farther. If I held such an opinion of the pathology of fever, I would treat the case as a local disease, by relieving the local inflammation, which is the cause of the vitiated secretions.—*Charleston Medical Journal.*

